# Oblique decision trees for spatial pattern detection: optimal algorithm and application to malaria risk

**DOI:** 10.1186/1471-2288-5-22

**Published:** 2005-07-18

**Authors:** Jean Gaudart, Belco Poudiougou, Stéphane Ranque, Ogobara Doumbo

**Affiliations:** 1 Medical Statistics and Informatics Research Team, LIF -UMR 6166 – CNRS/ Aix-Marseille University, Faculty of Medicine, 27 Bd Jean Moulin 13385 Marseille Cedex 05, France; 2 Immunology and Genetic of Parasitic Diseases, UMR 399 – INSERM/ Aix-Marseille University, Faculty of Medicine, 27 Bd Jean Moulin 13385 Marseille Cedex 05, France; 3 Malaria Research and Training Centre, Faculty of Medicine, Pharmacy and Odonto-Stomatology, University of Mali, BP 1805, Bamako, Mali

## Abstract

**Background:**

In order to detect potential disease clusters where a putative source cannot be specified, classical procedures scan the geographical area with circular windows through a specified grid imposed to the map. However, the choice of the windows' shapes, sizes and centers is critical and different choices may not provide exactly the same results.

The aim of our work was to use an Oblique Decision Tree model (ODT) which provides potential clusters without pre-specifying shapes, sizes or centers. For this purpose, we have developed an ODT-algorithm to find an oblique partition of the space defined by the geographic coordinates.

**Methods:**

ODT is based on the classification and regression tree (CART). As CART finds out rectangular partitions of the covariate space, ODT provides oblique partitions maximizing the interclass variance of the independent variable. Since it is a NP-Hard problem in R^N^, classical ODT-algorithms use evolutionary procedures or heuristics. We have developed an optimal ODT-algorithm in R^2^, based on the directions defined by each couple of point locations. This partition provided potential clusters which can be tested with Monte-Carlo inference.

We applied the ODT-model to a dataset in order to identify potential high risk clusters of malaria in a village in Western Africa during the dry season. The ODT results were compared with those of the Kulldorff' s SaTScan™.

**Results:**

The ODT procedure provided four classes of risk of infection. In the first high risk class 60%, 95% confidence interval (CI95%) [52.22–67.55], of the children was infected. Monte-Carlo inference showed that the spatial pattern issued from the ODT-model was significant (p < 0.0001).

Satscan results yielded one significant cluster where the risk of disease was high with an infectious rate of 54.21%, CI95% [47.51–60.75]. Obviously, his center was located within the first high risk ODT class. Both procedures provided similar results identifying a high risk cluster in the western part of the village where a mosquito breeding point was located.

**Conclusion:**

ODT-models improve the classical scanning procedures by detecting potential disease clusters independently of any specification of the shapes, sizes or centers of the clusters.

## Background

Since the development of warning systems and environmental hazards awareness, a wide range of statistical methods has been provided to identify disease clusters and spatial patterns. These methods have been classified into three groups [1-3]:

- Tests for focused clustering where the putative source is prespecified [4,5,2];

- Tests for global clustering with statistics using the distance between cases [6-9];

- General tests for localized clusters where the putative source or potential clusters cannot be prespecified [6,10,11].

This paper focuses on the latter tests i.e. on general procedures for the determination of spatial patterns. These patterns allow us to localize disease clusters where the disease rate is particularly high. Since the Openshaw's Geographical Analysis Machine (GAM), numerous works have proposed extensions or modifications of this method. The GAM lays out a regular grid of points covering the region under study. Then it generates overlapping circular windows centered at each grid point with constant radii depending on the grid spacing. The procedure is repeated at different predetermined values of the radius and thus defines potential clusters. Alternative procedures use circular windows centered at the observed point locations [10] and scan the area through this irregular grid. The use of squared shaped windows has also been proposed [6]. A general review of spatial methods is provided by Waller and Gotway [12] as well as in several publications [13-15].

The Kulldorff's scan statistic is one of the most interesting and used methods for cluster analysis [16,1,17]. The scan statistic is a likelihood ratio based method, which Kulldorff [11] defined without any assumptions about the shape, size or collection of locations for the scanning windows. However, various algorithms are necessary to calculate the test statistics for different defined types of scanning windows. Softwares (e.g. SaTScan™ [18]) have been implemented for some of these particular windows/algorithms. The SaTScan™ imposes on the map circular windows positioned on regular (such as GAM) or irregular grid (defined by the observed point locations). For each center point, the radius varies continuously from zero to a pre-specified upper bound. Each of the circular windows, moving through the different centers and with different radii, is a possible candidate for containing a cluster of cases.

It is noteworthy that the detection of potential clusters is enforced on circular shaped (or squared shaped) windows. The various algorithms applied the scan statistic method to windows centered at either grid or observed point locations. These two procedures define different sets of potential clusters and therefore may not provide exactly the same results. Furthermore, changing the windows' shape may also provide different clusters. Gangnon and Clayton introduced a bayesian approach [19] for clustering which does not require cluster's locations or shapes to be specified but which requires some prior specifications of the distribution of various cluster size and shapes (hierarchical priors). However, given the large number of potential models, the posterior distribution cannot be directly provided. Therefore, Gangnon and Clayton limit the number of models under consideration by using a randomized method to build models with high posterior distributions. They approximate the posterior distribution over the limited number of cluster models incorporating hierarchical prior. Patil and Taillie [20] proposed an adaptation of the scan statistics to detect clusters without restricted shape. It reduces the size of the potential cluster set by determining levels of the rates of cases. The potential cluster set consists on all the connected components that have rates higher than a fixed level. Each level determines a potential cluster set. But the determination of levels is data-dependent. Furthermore in a practical point of view, not all of the observed rates can be used as levels in order to avoid providing a computationally impracticable number of potential cluster sets. Other procedures use stochastic optimization algorithm to reduce the number of examined potential clusters [21]. But again these methods used for the determination of potential clusters are not optimal from a classification viewpoint.

The aim of the present work is to provide an optimal partitioning procedure using Oblique Decision Trees in order to detect spatial patterns and to optimize the potential clusters determination without prior specifications. Rather than using a likelihood ratio test, this new approach, which is not a scan statistic, is based on the calculus of the interclass variance during each of many splits of the space before providing the final pattern.

## Methods

### CART and ODT-models

Tree-based models such as CART (Classification And Regression Trees) [22] are non-linear and non-parametric alternatives to linear models for regression and classification problems (such as linear regression, logistic regression, linear discriminant analysis, linear proportional hazard models). CART models are fitted by binary recursive partitioning of a multidimensional covariate space, in which the dataset is successively split into increasingly homogeneous subsets until a specified criterion is satisfied. For the first partition, CART searches the best possible place to split a continuous variable into two classes and defines two subspaces which maximize overall class separation (i.e. interclass variance of the dependent variable). Each of these subspaces subsequently serves as the basis for further partitioning independently of the others and so on. At each step the variable used for each split is selected from all the explicative variables so as to provide an optimal partition given the previous actions. Partitions' sequence is summarized by a binary tree. The root node of tree corresponds to the entire data space. Partitions of the space are associated with descendants of the root node. The leaves of the tree, or terminal nodes, correspond to subspaces which are not further partitioned. The stability of the procedure can be improved using Data resampling.

While CART-models are widely used as exploratory techniques they are less-commonly used for prediction. Trees generally rely on fewer assumptions than classical methods and handle a wide variety of data structures. Furthermore they are easy to use and to interpretate, and thus provide a wide range of application fields. The use of CART procedure has been considered by others in a variety of medical problems [22,23] such as, for example, survival analysis [24-26], longitudinal analysis, diagnostic and prognostic studies or clinical trials [27-30].

One particular application is signal processing [31], in which the problem concerns the detection of multiple change points in the mean. The CART procedure can be used to estimate simultaneously the change-points and the means by recovering an underlying piecewise constant function ***f***(***t***).

If ***m*_*k *_**are the means for each piecewise ***k***, then ***t*_*k *_**are the change-points:



If we extend this point of view to the covariate space defined by geographic coordinates, CART estimates the "change-lines" (instead of change-points) of a piecewise constant function on R^2^. In other words, tree-based procedure can easily determine spatial patterns.

However, one limitation is that CART provides axis-parallel splits i.e. rectangular spatial patterns. Oblique decision trees (ODT) deal with this problem. Those algorithms produce oblique (and then polygonal) partitioning of the covariate space. However, oblique trees are less popular than axis-parallel trees because the splits are less straightforward to interpret and oblique procedures require greater computational complexity than axis-parallel algorithms. Finding the best oblique tree in the covariate space is a NP-Hard problem [32]. Therefore, existing ODT algorithms use deterministic heuristics or evolutionary algorithms (like the OC1 system [33]) to find appropriate hyperplanes for partitioning the covariate space [22,34,32,33]. Comparisons of the different procedures are provided, for example, by Murthy [33] Cantu-Paz [34] and Bradley [35].

Despite this difficulty in R^N^, it is easier to find an oblique partition in the particular case of a space determined by the geographic coordinates, i.e. in R^2^. Evolutionnary or heuristic algorithms are not robust. They provide occasionally local minima [33] and therefore are not optimal procedures in R^2^. The ODT-algorithm we have developped is an optimal procedure to reach an optimal solution without using heuristics or evolutionary procedures.

### ODT algorithm

The general purpose of the entire procedure consists on finding several partitions of the plane. We present the first step which allows finding the best oblique split of the plane. Going recursively, this algorithm will split the plane into several partitions, until reaching a specific criterion.

This subsection is organized as follow:

i. First, we will introduce how the plane is splitted into two adjacent partitions according to the interclass variance.

ii. Second, we will present how the finite set of oblique lines is determined, still within the first step of the entire procedure.

iii. Third, we will propose an optimization of this algorithm.

**i. **The splitting method proceeds as follows.

Consider, in the geographical space represented by the plane with an orthogonal basis {***x***, ***y***} and a fixed origin ***O***, ***n ***points ***M*_*i *_**with coordinates {***x*_*i*_**, ***y*_*i*_**}. These coordinates can represente the geographic coordinates of a point location provided by GPS. To each point ***M*_*i *_**a numeric random variable ***Z*_*i *_**(called explained or dependant variable) is associated with the observation ***z*_*i *_**Whereas the CART procedure partitions the plane according to a line parallel to the axis maximizing the interclass variance of ***z*_*i*_**, our procedure partitions the plane according to an oblique line  maximizing in the same way the interclass variance of ***z*_*i*_**.

To find this oblique line according to the direction  we have to define the perpendicular direction ***u ***and the angle .

From a general viewpoint, for a fixed direction  the procedure has to:

- Orthogonally projects the points ***M*_*i *_**on the (***O***, ***u***) direction, defining the coordinate ***u*_*i*_**;

- Considers all the ***u*_*i *_**as potential threshold in the way to split the plane with the direction  perpendicular to the direction ***u ***and going through ***u*_*i*_**;

- Finds the optimal split between two adjacent classes, maximizing the interclass variance of ***z*_*i *_**according to theses projections.

**ii. **The splitting method provides a finite set of cluster proceeding as follows.

Before detailing the algorithm, we have to study the different splitting directions  i.e. to specify wich angles *θ *have to be analyzed. For a global solution the algorithm can scan all the oblique directions (i.e. all the *θ*) between zero and *π*. In a heuristic way one can also discretize this interval providing a finite number of angles *θ*. But these two methods are not optimal.

**Figure 1:**
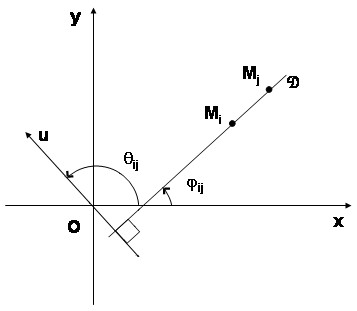
**Construction of the critical angle *θ*_*ij *_of the direction *u***. - the geographical space is represented by the plane with an orthogonal basis {***x***, ***y***} and a fixed origin ***O***; - ***u ***is a direction perpendicular to the splitting direction ; - ***M*_*i *_**and ***M*_*j *_**are two point locations in the geographical space.

The optimal algorithm for an optimal solution is easy to implement. Obviously, two points ***M*_*i *_**(***x*_*i*_**, ***y*_*i*_**) and ***M*_*j *_**(***x*_*j*_**, ***y*_*j*_**) have the same projected coordinates on the (***O***, ***u***) direction if and only if ***M*_*i *_*M*_*j *_**is perpendicular to (***O***, ***u***) (Figure 1). Then the number of critical directions, defined by the *θ*_*ij *_angles, exists and is finite.

For each direction  passing through two points ***M*_*i *_**(***x*_*i*_**, ***y*_*i*_**) and ***M*_*j *_**(***x*_*j*_**, ***y*_*j*_**), we define *φ*_*ij *_the angle between the line ***M*_*i *_*M*_*j *_**and the x-axis.

Then:



As previously defined, *θ *is the angle between the x-axis and the direction (***O***, ***u***) perpendicular to ***M*_*i *_*M*_*j*_**.

Then for each couple (***M*_*i*_**, ***M*_*j*_**), we have 

Each critical angle *θ*_*ij *_defines an angular sector. Within each sector, the order of the coordinates projected on the (***O***, ***u***) direction does not depend on this direction. For points ***M*_*i *_**and ***M*_*j *_**the difference (***u*_*j *_**- ***u*_*i*_**) of their coordinates projected on (***O***, ***u***) verifies:

(***u*_*j *_**- ***u*_*i*_**) ***cos***(*φ*_*ij*_) = (***x*_*j *_**- ***x*_*i*_**) ***sin***(*θ *- *θ*_*ij*_)     **(1)**

with (***u*_*j *_**- ***u*_*i*_**) = (***y*_*j *_**- ***y*_*i*_**) ***sin***(*θ*) for ***x*_*i *_**= ***x*_*j *_**⇔ *φ*_*ij *_= 

Thus (***u*_*j *_**- ***u*_*i*_**) depends continuously on *θ*. The sign of this difference cannot change within the angular sector since (***u*_*j *_**- ***u*_*i*_**) = 0 only if *θ *= *θ*_*ij*_.

**Figure 2:**
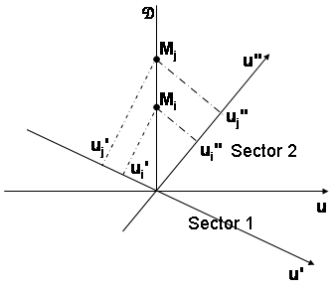
**Passage through the critical direction *u*, from sector 1 to sector 2**. - ***u ***is a direction perpendicular to the splitting direction ; ***M*_*i *_**and ***M*_*j *_**are two point locations in the geographical space; - Change in the order of the projected coordinates on the ***u' ***and ***u" ***directions; - ***u' ***and ***u" ***are directions with intermediate angles, belonging respectively to sector 1 and sector 2; - ***u'*_*i*_**, ***u'*_*j*_**, ***u"*_*i*_**, and ***u"*_*j *_**are the projected coordinates of points ***M*_*i *_**and ***M*_*j*_**: ***u'*_*i *_**> ***u'*_*j *_**and ***u"*_*i *_**<***u"*_*j*_**.

It follows that the interclass variances (and then the ODT procedure) is not modified within each sector. As a direct consequence of **(1) **the transition from a sector to another via the critical angle *θ*_*ij *_(Figure 2) induces the same order except the permutation of the two adjacent elements (***u*_*i*_**, ***u*_*j*_**).

Note that for aligned points ***M*_*i*_**, ***M*_*j *_**and ***M*_*k *_**the algorithm has to permute the adjacent element group (***u*_*i*_**, ***u*_*j*_**, ***u*_*k*_**). Similarly for parallel directions ***M*_*i *_*M*_*j *_**and ***M*_*k *_*M*_*l*_**, the algorithm has to permute at the same time the couples of adjacent elements (***u*_*i*_**, ***u*_*j*_**) and (***u*_*k*_**, ***u*_*l*_**).

Note again that all these angular sectors define as much covariates. Thus the procedure comes to the usual CART procedure. But the number of different critical angles is  and using CART this way over-consumes time and space. For example, in our application the number of point locations is ***n ***= 164, hence the number of different angular sectors is ***N ***= 13270.

**iii. **We present now an optimization of our algorithm. A less time consuming and more efficient algorithm is a stepwise analysis of the angular sector, ordered according to the observed *θ*_*ij*_. At each step the algorithm uses the previous calculus.

Because only two elements between two adjacent sectors are permuted only one interclass variance has to be reloaded, related to the single different split (or some interclass variances for the group of permuted couples, related to a few different splits). The procedure inherits the calculus of the other interclass variances from the previous sector with the exception of the interclass variance related to the single permutation.

Thus, the algorithm complexity is (***n***^2 ^***log n***) in time and (***n***) in space for one split. Finally, our algorithm splits the plane into two adjacent partitions as follows:

• Arrange the ***x*_*i*_**;

• Calculate and arrange the *θ*_*ij *_via the ***a*_*ij*_**;

• Calculate ;

• For each potential split of the first angular sector (corresponding to the x-axis), i.e. for each value of ***x*_*i*_**:

- Calculate the ∑ ***z*_*i *_**for each class (on both sides of the threshold ***x*_*i*_**) and then the interclass variance, using the previous results;

- If the calculated interclass variance is greater than the previous one, store the results;

• For the next angular sector

- Permute the corresponding ***x*_*i *_*x*_*j *_**(or the group of elements);

- Calculate the ∑ ***z*_*i *_**only for the two classes generated by the split between ***x*_*j *_**and ***x*_*i *_**(or some splits for the group of permuted elements);

- If the new interclass variance is greater than the previous optimum, store the results;

• Until all sectors are scanned.

This algorithm goes on recursively until a specific criterion is reached and the Oblique Decision Tree is completed.

For simplicity we will not herein discuss special procedures of CART such as stopping rules, pruning algorithms or resampling methods; these are examined elsewhere [22,36].

### Dataset

Malaria is the major parasitic disease in the world affecting approximately 300–500 million individuals annually. About two percents of the individuals infected with *Plasmodium falciparum *die. Most of the deaths occur in children. In the last decade, the incidence of malaria has been increasing at an alarming rate in Africa representing over 90% of the reported cases in the world [37].

The study area was the whole village of Bancoumana located in the administrative circle of Kati (Mali, Western Africa). This village is located in the high Niger's valley, a Sudanese savannah area, about 60 km south-west from the capital city Bamako. The main activities are rice cultures and truck farming along the Niger river. This village is 2.5 km^2 ^wide, with 8 000 inhabitants (MRTC census, 1998) and about 1 600 children under 9 years. The transmission of malaria is high during the rain season (usually from June to October, with temperatures varying between 25°C and 40°C). It decreases then, reaching a low level of transmission one or two months thereafter.

The project investigated at a village-level approach (using a 1–3 m resolution scale) the risk of malaria infection. The presence of *P. falciparum*, the main infectious agent of malaria in this area, in blood smears was investigated in 1 461 children living in 164 households during the dry season in March 2000. Among them, 474 children had a positive blood smear (32.44%, CI95% [30.09–34.89]). Localization was performed through GPS receivers. Thus, all children were geocoded at a point location (corresponding to their house). Geo-database and cartographic displays were provided with the ArcGIS 8.3 software (ESRI, Redlands, CA).

Human subjects' research conducted in these studies was approved by the Institutional Committee on Ethics of the Mali Faculty of Medicine and Pharmacy, University of Mali. To obtain informed consents a stepwise consent process was applied as described by Doumbo [38]. First, the community informed consent was obtained before the beginning of the study. Second, the informed consent of the parents or guardians of the children were orally obtained before each clinical or biological investigation.

### Data analysis

The ODT-algorithm was implemented with the Matlab Software 7.0.1 (The Mathworks Inc. 2004). We applied the ODT procedure to the dataset using the GPS coordinates of each location as independent covariates and the parasitic positivity rate (rate of positive blood smears per houses) as dependant variable. Thus ODT provided an optimal partition of the geographical area, i.e. a spatial pattern of the disease risk. We chose to use two classical stopping rules [22]. First, the ODT stopped if a class was made up of less than 15 locations. Second, we prunned a node if, after partition, one of the two resulting classes was made up of less than 3 locations.

For inference we considered the constant risk hypothesis as a model of "no pattern". Under this null hypothesis each child is at the same disease risk within the observation period regardless of his location. Thus the classes issued from the ODT displayed similar disease risk. However in keeping with many spatial health applications [12], we can not rely on asymptotic arguments to derive theoretically the associated distributions under the null hypothesis. Monte Carlo (MC) simulations were flexible tools for such assessment.

Similarly to many statistical models, we used for inference the explained variability rate R_v_, defined as the ratio of the interclass sum of squared errors (SCE) (outcome of the ODT model) and the total SCE. We considered a Monte Carlo inference conditional on the set of all locations and on the local number of subjects. The total number of cases varied from simulation to simulation with an expected value (the total number of cases on the observed dataset). In this way, the simulations assessed spatial variations in the local proportion of cases conditional on the set of all locations. Monte Carlo simulations reflected a constant risk hypothesis similarly to the Rushton and Lolonis [39] approach. We ran 999 simulations under the constant risk hypothesis i.e. homogeneous Poisson distribution. Under this null hypothesis we applied the ODT-algorithm for each of the random dataset and calculated the empirical distribution of R_v_. Thus the MC inference provided p-values for testing whether or not the observed explained variability rate is a realization of the theoretical (simulated) distribution under the constant risk hypothesis. In other words, MC inference tested the ODT-model and provided the significance of the spatial pattern issued from the oblique decision tree.

We compared the ODT-model outputs with those of the scan statistic method. For the latter, we used the software program SaTScan™ [18] in order to test for the presence of spatial clusters of malaria infection and to estimate their locations. The identification of high risk clusters with the SaTScan™ was performed under the Poisson probability model assumption using a maximal cluster size of 50% of the total population. For statistical inference, 999 Monte Carlo replications were performed. The null hypothesis of no clustering was rejected when the simulated p-value was lower than or equal to 0.05.

During the data analysis we calculated all confidence intervals of rates according to the Wilson method [40].

## Results

### Oblique Decision Tree

**Figure 3:**
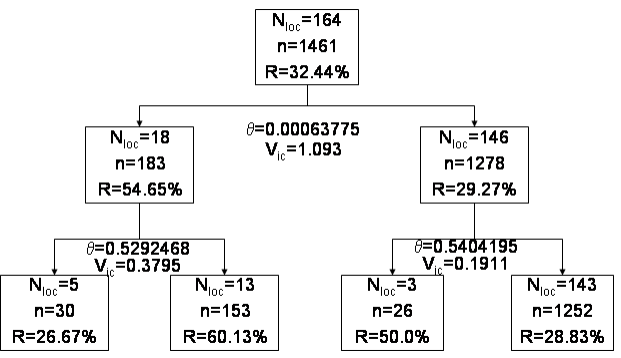
**Oblique Decision Tree for spatial partitioning**. The geographical area is splited into 6 partitions. *N*_*loc*_: number of locations belonging to each partition; *n*: total number of children of each partition; *R*: infectious rate; *θ*: critical angle for each split; *V*_*ic*_: interclasses variance for each split.

**Figure 5:**
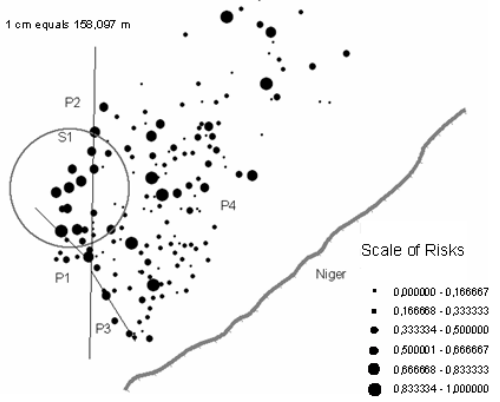
**The village of Bancoumana**. - The circle **S1 **refers to the significative cluster provided by the Kulldorff's SaTScan. - The strait lines are the 3 splits resulting from the ODT-model, providing 4 partitions **P1**, **P2**, **P3 **and **P4**. - The bold grey line represents the Niger river. - Each location is represented by its own risk value. The scale of risks is discretized in 6 equal sized intervals.

The ODT (Figure 3) partitioned the village into four risk classes. The explained variability rate is high, i.e. R_v _= 83.96% of the variability is explained by the ODT-model. The global risk of disease (Table 1) was 32.44%, CI95% [30.09–34.89]. The ODT provided two classes of high infection risk. In the first high risk class (P2), located in the western part of the village (Figure 5), the risk was 60%, CI95% [52.22–67.55]. In the second high risk class (P3), located in the southern part of the village, the risk was about 50% with a large confidence interval. Note that during the rain season about 80% of the children had a positive blood smear in the whole village. Investigations at this site pointed to a small pond located within the western high risk class, and to ricefields located in the southern part of the village, both having been identified as *Anopheles *(the vector of malaria) breeding places.

**Table 1:** Spatial pattern resulting from the ODT-model. The first line refers to the areas without any partition.

	Centroid's Coordinates^a^	Pop.^b^	Risk of infection [CI95%]	Number of Locations^c^
No pattern	X = -8.266497256 Y = 12.20520982	1 461	32.44% [30.09–34.89]	164
P1	X = -8.270634 Y = 12.202594	30	26.67% [14.18–44.45]	5
P2	X = -8.27019 Y = 12.20438615	153	60.13% [52.22–67.55]	13
P3	X = -8.26849 Y = 12.1999733	26	50.0% [32.06–67.94]	3
P4	X = -8,2659751 Y = 12.205486	1 252	28.83% [26.39–31.4]	143

a- The coordinates are for the centroid of each partition.

b- Pop. refers to the total number of children included in each partition.

c- The number of locations refers to the total number of households within each partition.

**Figure 4:**
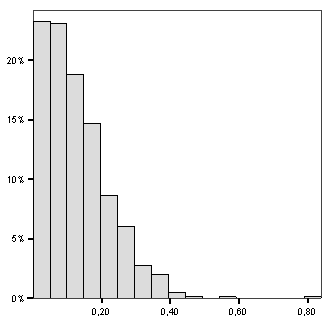
**Empirical distribution of the explained variability rate R_v_**. The distribution was provided by Monte Carlo procedure (999 simulated sets and one observed set).

Monte Carlo inference provided a global test, testing the null hypothesis of a homogeneous Poisson distribution of the malaria infection cases within the study area. Under this null hypothesis we provided (999 simulated sets and one observed set) the empirical distribution of the explicated variability rate R_v _(Figure 4). In this application the R_v _provided by the ODT-model significantly differed (p < 0.0001) from the one provided under the homogeneous Poisson distribution, i.e. the spatial pattern was significant.

### Satscan approach

The Satscan results yielded one significant clusters (Table 2). In the first cluster (**S1**) the risk of disease was high with an infectious rate of 54.21% (CI95% [47.51–60.75]). Obvioulsy, his center was located within the high risk ODT partition (**P2**) and the risk of disease were similar in S1 and P2 (Figure 5). The second and third high clusters were not significant, totalizing only one point location each.

**Table 2:** High risk of malaria spatial clusters in Bancoumana, Mali, march 2000.

	Cluster						
						
	Coordinates^a^	Radius Km	Pop^b^	Risk of infection [CI95%]	Cases Obs/exp	Loc^c^	*p*^d^
S1	X = -8.27047 Y = 12.205325	0.27	214	54.21% [47.51–60.75]	Obs:116 Exp:69.43	22	0.001
S2	X = -8.26701 Y = 12.205729	0.00	7	85.71% [48.69–97.43]	Obs:6 Exp:2.27	1	0.998
S3	X = -8.26469 Y = 12.207877	0.00	20	60.00% [38.66–78.12]	Obs:12 Exp:6.49	1	0.999

a- The coordinates are for the center of each circle.

b- Pop. refers to the total number of children into each cluster.

c- Loc. refers to the number of locations belonging to each cluster.

d- p-values refer to the Monte Carlo inference, after 999 replicates.

## Discussion

For spatial cluster detection, the specification of the shape and size of the clusters is required rather than using political or administrative definitions of zones. For this purpose scanning methods provide sets of potential clusters but the problem of the choice of the shape still remains. Different scanning grid and different windows' shapes or sizes may provide different sets of potential clusters. To reduce this difficulty we introduced ODT-models with the aim to detect spatial pattern without pre-specifying windows' shape. In contrast to classical scanning procedures, neither the shape, size, nor centroid location have to be specified by the users. Thus, ODT are optimal procedures from the classification viewpoint.

Furthermore the spatial pattern obtained by the ODT-model defines a potential clusters set which can then be tested using the classical Monte Carlo inference. Similarly to Satscan, inference analysis has to avoid multiple testing inherent to such a procedure. The Kulldorffs procedure provides first a potential cluster set. Second, this procedure performs a significance test based on the local likelihood ratio statistic for each cluster in a way that compensates for the multiple testing. In our work we provide a global inference, testing the significativity of the spatial pattern obtained by ODT. Note that, similarly to Kulldorff's inference, likelihood ratio tests can be used to test the spatial pattern.

Recently, Tango proposed a flexibly spatial scan statistic to detect noncircular clusters [41]. But the Tango's method is not practically feasible for large clusters (more than 30 point locations).

Our findings indicate that the ODT-method is consistent with the classical Kulldorf's scan statistic. ODT procedure is thus a classification tool widely usable for spatial pattern detection. When compared to ODT, the scan statistic did not detect the second high risk cluster (P3). This is probably due to the lack of points fitting in this cluster (3 point locations and 26 childrens). The 95% confidence interval of the disease rate in this cluster is large (32.06%-67.94%). Nevertheless, after investigations in the village, a putative source of disease risk has been detected at this location. The two non-significant high risk clusters (S2 and S3) enclose only one point location each. This might explain both the lack of significativity with the satscan method and the lack of detection by the ODT.

After detection of a significant spatial pattern, the next logical step is to test whether this pattern can be explained by known or suspected risk factors. For example in the context of malaria, environmental factors such as mosquitoes breeding sites or thatched habitations might be identified and appropriate measures can be proposed to enhance the disease's control policy.

ODT-models allow for a flexible relationship between the variables. The relationships between covariates do not need to be linear or additive and the interactions do not need to be prespecified or to be of a particular multiplicative form. The literature about tree-based models is increasing particularly for studies focusing on formal inference procedures [31,36]. In contrast to classical ODT-procedures the algorithm herein described is optimal since it uses neither evolutionary algorithms [32,34] nor heuristics [22]. While the problem is NP-Hard in R^N^, the algorithm remains polynomial in R^2^.

The stability of tree-models can be improved by resampling methods. It is noteworthy that scanning methods such as satscan can also benefit from resampling. Resampling methods may improve the determination of the potential cluster set when the SaTScan™ procedure uses windows centered at each point location. Among different stopping rules, criteria have to be chosen according to the trade-off between variance and bias of prediction. The usually chosen rules are known as flexible and robust methods [31]. But as our application results indicate, less restrictive rule can be used for specific epidemiological dataset in order to improve the interpretation of the ODT-models' output. For rare diseases it might be necessary to use less stringent stopping rules than for diseases characterized by an epidemic evolution. This is related to the definition of "cluster of cases" which depends on the epidemiological profile of the disease.

The risk of infection has a high geographic variability [42,43] and the knowledge of this variability is essential to enhence malaria control programs' efficiency [44]. Moreover, the detection of high-risk locations is one recommendation of the 20^th ^WHO technical report [45]. In this context, the development of GIS displays data on local malaria cases and then stratification of malaria risk providing the opportunity for more focal (and then efficient) malaria control programs [42].

## Conclusion

In conclusion, Oblique Decision Tree is a new approach for spatial pattern detection and has the following features:

- ODT improve the classical scanning procedures by providing polygonal potential clusters;

- ODT are not bound by fixed centroid locations, sizes or shapes. Thus, first they have an enhanced flexibility. Second, the results are independent from the shape size and center pre-specification;

- ODT provide an optimal partition in the classification viewpoint.

Thus, ODT-models favorably compare with other cluster detection methods for spatial epidemiology.

## Abbreviations

CART: Classification And Regression Tree

CI95%: 95% Confidence Interval

GIS: Geographic Information System

GPS: Global Positioning System

MC: Monte Carlo

MRTC: Malaria Research and Training Center, Bamako, Mali.

ODT: Oblique Decision Tree

SCE: sum of squared errors

## Competing interests

The author(s) declare that they have no competing interests.

## Authors' contributions

J Gaudart provided the ODT procedure, implemented the algorithm, performed statistical and geographical analysis, and drafted the manuscript.

B Poudiougou, S Ranque and O Doumbo carried out the epidemiological georeferenced data and provided the parasitological analysis.

O Doumbo initiated and supervised the epidemiological study.

All authors wrote and approved the final manuscript.

## Pre-publication history

The pre-publication history for this paper can be accessed here:


